# Supplementary material for: Efficient and Selective Oxygenation of Cycloalkanes and Alkyl Aromatics with Oxygen through Synergistic Catalysis of Bimetallic Active Centers in Two-Dimensional Metal-Organic Frameworks Based on Metalloporphyrins
Source: Biomimetics (Basel). 2023 Jul 21;8(3):325. doi: 10.3390/biomimetics8030325 (PMC10807029; doi:10.3390/biomimetics8030325)
Supplement: Supplementary file 1 [file biomimetics-08-00325-s001.zip › biomimetics-2495525-supplementary.pdf]

Supplementary Information

Efficient and Selective Oxygenation of Cycloalkanes and Alkyl Aromatics with Oxygen through Synergistic Catalysis of Bimetallic Active Centers in Two-Dimensional Metal-Organic Frameworks Based on Metalloporphyrins

Xin-Yan Zhou, Bo Fu, Wen-Dong Jin, Xiong Wang, Ke-Ke Wang, Mei Wang, Yuan-Bin She and Hai-Min Shen*

College of Chemical Engineering, Zhejiang University of Technology, Hangzhou 310014, China

1. Experimental section

2. Experimental data

3. Spectra of metalloporphyrins characterization

4. Spectra of radical capture

5. References

* Correspondence: haimshen@zjut.edu.cn; Tel.: +86-15988460002

1. Experimental section

1.1 Chemicals and materials

Pyrrole (99%), methyl 4-formylbenzoate (98%), anhydrous copper(II) acetate (98%), anhydrous zinc(II) acetate (98%) and anhydrous cobalt(II) acetate (98%) utilized in metalloporphyrin syntheses were purchased from Energy Chemical Co., Ltd., shanghai Lingfeng Chemical Reagent Co., Ltd., and Adamas Reagent Co., Ltd., respectively. Copper(II) nitrate trihydrate (99%), zinc(II) nitrate hexahydrate (99%), cobalt(II) nitrate hexahydrate (99%), nitric acid (65%~68%) and N, N-dimethylformamide (analytical pure) were purchased from Sinopharm Chemical Reagent Co., Ltd., Adamas Reagent Co., Ltd., and Shanghai Aladdin Biochemical Technology Co., Ltd., respectively. Cycloalkanes and aromatics in their highest purities available employed as substrates in catalytic oxidation were the commodities of Aladdin Biochemical Technology Co., Ltd., Sinopharm Chemical Reagent Co., Ltd., Macklin Biochemical Technology Co., Ltd., and Adamas Reagent Co., Ltd., respectively. Cycloalkanols, cycloalkanones, aromatic alcohols, aromatic ketones and carboxylic acids utilized as standard samples in GC and HPLC analyses were purchased from Alfa Aesar, TCI, Meryer and Macklin respectively. Common reagents in the experiments were purchased from Hangzhou Shuanglin Chemical Reagent Co., Ltd., Sinopharm Chemical Reagent Co., Ltd., Guangdong Guanghua Sci-Tech Co., Ltd., and Shanghai Lingfeng Chemical Reagent Co., Ltd., respectively and used directly without any purification.

1.2 Characterizations and instruments

The ^{1}H NMR and ^{13}C NMR spectra were collected on a Bruker AVANCE III (500 MHz) spectrometer employing tetramethylsilane (TMS) as internal standard. The ESI-MS spectra were recorded on an Agilent 6210 LC/TOF mass spectrometer equipped with electrospray ionization source. The FT-IR spectra were measured on a Thermo Nicolet 6700 spectrometer. The XPS analyses were conducted on a Kratos AXIS Ultra DLD spectrometer. The SEM images were recorded on a Zeiss Gemini 500 microscope. The TEM images were collected on a Hitachi HT 7700 microscope. The EDS analyses were carried out on an energy dispersive spectrometer coupled with the SEM and TEM analyses. The TG analyses were performed on a PerkinElmer Diamond TG/DTA instrument under air atmosphere. The XRD patterns were collected on a PNAlytical X'Pert Pro X-ray diffractometer. The N_2 adsorption-desorption isotherms were measured on a Micromeritics ASAP 2460 instrument at -196 °C. And the BET specific surface area was obtained from the above isotherms. The pore size distribution was determined using the NLDFT model. The EPR analyses were conducted on a JEOL JES-FA200 spectrometer to investigate the free radical species in the catalytic system. And 5,5-dimethyl-1-pyrroline N-Oxide (DMPO) was used as the radical trapping agent. The GC analyses were carried out with a TG-5MS capillary column (30 m × 0.32 mm × 0.25 μm) on a Thermo Scientific Trace 1300 instrument. The HPLC analyses were conducted with an Amethyst C18-H chromatography column (150 mm × 4.6 mm× 0.25 μm) on a Thermo Scientific Ultimate 3000 chromatography equipped with an Ultimate 3000 Photodiode Array Detector.

1.3 Synthesis of tetrakis(4-methoxycarbonylphenyl)porphyrin ($T(4\text{-}COOCH_3)PP$)

The tetrakis(4-methoxycarbonylphenyl)porphyrin($T(4\text{-}COOCH_3)PP$) was produced using the procedure described in the literature with minor modifications[1, 2]. Under the protection of nitrogen, methyl 4-formylbenzoate (82.08 g, 500 mmol) was dissolved in propionic acid (1500 mL) in a four-neck flask (2.0 L), and the resulting solution was agitated and heated to refluxing in the dark. In around 15.0 minutes, freshly distilled pyrrole (33.55 g, 500 mmol) was added dropwise, and the resulting black liquid was stirred under reflux for 2.0 hours. The purple material was dissolved in dichloromethane (100 mL) and put into methanol after suction filtering under decreased pressure (500 mL). After allowing the collected precipitate to remain at room temperature for 8.0 hours, it was dissolved in dichloromethane (100 mL), poured into methanol (500 mL), and filtered two more times. The purple material was then purified using silica column chromatography with acetone and chloroform as the eluent (Vacetone: V chloroform = 1: 100). The desired product was achieved as purple crystal (20.26 g) with a yield of 19.14% after being dried at 70 °C for 5.0 hours under reduced pressure. ^1H NMR (500 MHz, $CDCl_3$): δ = 8.82 (s, 8H), 8.44 (d, 8H), 8.30 (d, 8H), 4.11 (s, 12H), -2.81 (s, 2H). ^{13}C NMR (125 MHz, $CDCl_3$): δ = 167.26, 146.65, 134.54, 129.82, 128.02, 119.42, 52.47. ESI-MS(m/z): 847.2 $[M+H]^+$, 869.2 $[M+Na]^+$.

1.4 Syntheses of tetrakis(4-methoxycarbonylphenyl)porphyrin metal(II) ($T(4\text{-}COOCH_3)PPM$)

The tetrakis(4-methoxycarbonylphenyl)porphyrin metal(II) (T(4-$COOCH_3$)PPM) was produced using the procedure described in the literature with minor modifications [1-3]. In the synthesis of T(4-$COOCH_3$)PPCo, for example, T(4-$COOCH_3$)PP (3.3876 g, 4.0 mmol) and anhydrous cobalt(II) acetate (7.0808 g, 40.0 mmol) were mixed in N, N-dimethylformamide (600 mL) in a four-neck flask (1.0 L), and the resulting mixture was agitated with heating to refluxing After 12.0 hours of stirring at the refluxing temperature, the reaction mixture was cooled to room temperature and 300 mL of water was added. The suction filtration precipitate was dissolved in dichloromethane (100 mL) and rinsed twelve times with water (12 x 100 mL). Anhydrous sodium sulfate was used to dry the CH_2Cl_2 layer. The solvent was evaporated under low pressure, and the red power produced was purified further using silica column chromatography with dichloromethane as the eluent. A brownish red power (2.0924 g) was obtained as the goal product after drying at 70 °C for 5.0 hours under decreased pressure with a yield of 57.88%. ESI-MS(m/z): 903.2 $[M]^+$. The same to synthesize T(4-$COOCH_3$)PPCu and T(4-$COOCH_3$)PPZn.

1.5 Syntheses of tetrakis(4-carboxylphenyl)porphyrin metal(II) (T(4-COOH)PPM)

As shown in **Scheme S1**[1-3], the synthesis of tetrakis(4-carboxylphenyl)porphyrin metal(II) (T(4-COOH)PPM, TCPPM) was carried out. T(4-COOH)PPCo was synthesized by dissolving tetrakis(4-methoxycarbonylphenyl)porphyrin cobalt(II) (T(4-$COOCH_3$)PPCo) (2.7114

g, 3.0 mmol) and KOH (10.0998 g, 180 mmol) in a combination of methanol (100 mL), tetrahydrofuran (100 mL) and water (1000 mL), and the resultant mixture was heated to refluxing and kept stirring under refluxing for 12.0 h. The resulting solid was dissolved in water (500 mL) after the solvent was removed, and aqueous HCl (2.0 mol/L) was added to acidify the solution to pH = 2.0. Suction filtration precipitate was rinsed three times with water (3 x 200 mL) and dried at 70 °C for 5.0 hours under decreased pressure. The intended product was a red power (2.3448 g) with a yield of 92.20%. ESI-MS(m/z): 847.1 $[M]^+$. The same to synthesize T(4-COOH)PPNi、T(4-COOH)PPFe、T(4-COOH)PPMn.

1.6 Free radical trap research

Catalyst (Co-TCPPNi 8.0 mg, 0.08 mg/mmol), free radical trap reagent (bromochloroform ($CBrCl_3$), tert-butyl bromide ($(CH_3)_3CBr$) or diphenylamine (Ph_2NH), 5.0 mmol), and substrate (cyclohexane 8.4160 g, 100 mmol) were added to a 50 mL reactor. The reaction mixture was heated to temperature (120 °C) with stirring, and oxygen was injected in the reactor until the pressure reached 1.0 MPa. The resultant mixture was cooled to room temperature in cool water after 8.0 hours of stirring at temperature (130 °C) and pressure (1.0 MPa), then triphenyl phosphine (1.3115 g, 5 mmol) was added to transform the remaining peroxide. The resulting liquid was accurately diluted with acetone to 50 ml.

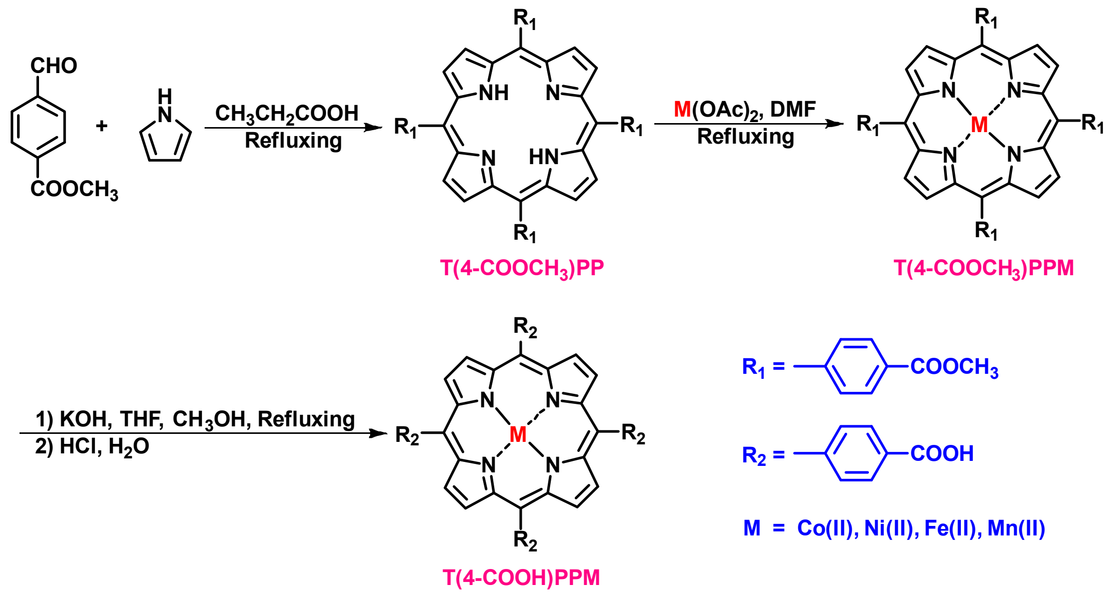

Scheme S1. Schematic syntheses of porphyrins and metalloporphyrins.

2. Experimental data

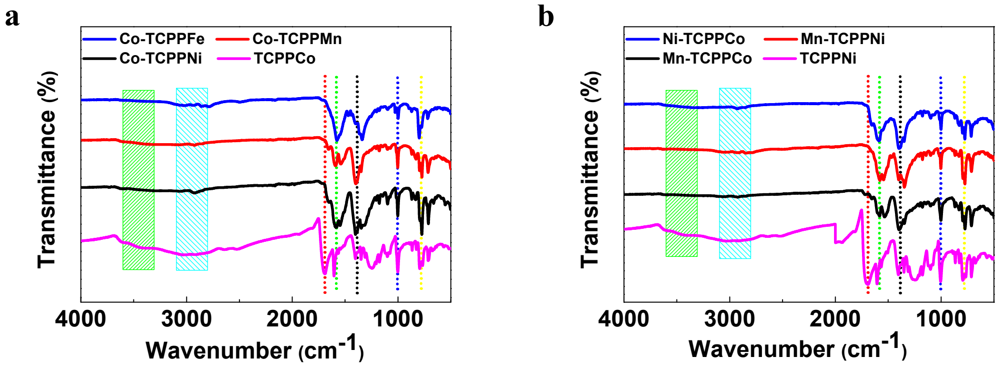

Figure S1. FT-IR spectra of the synthesized two-dimensional MOFs and corresponding metalloporphyrin.

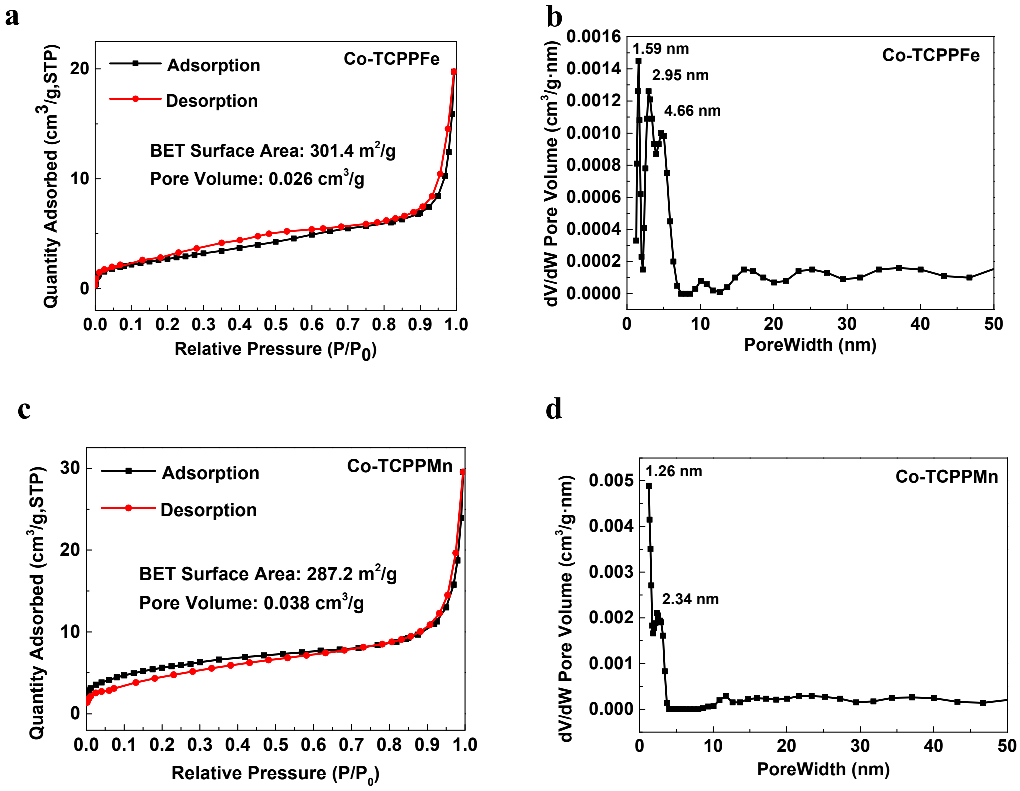

Figure S2. Nitrogen adsorption-desorption isotherms and pore width distributions of representative two-dimensional MOF Co-TCPPFe (a, b) and Co-TCPPMn (c, d).

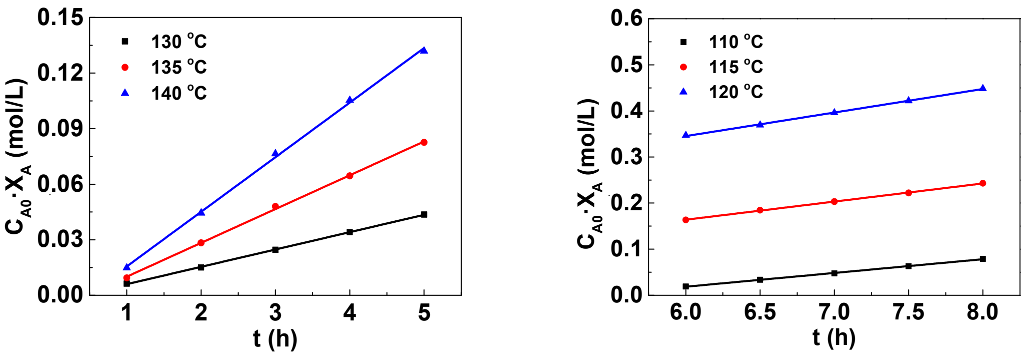

a b

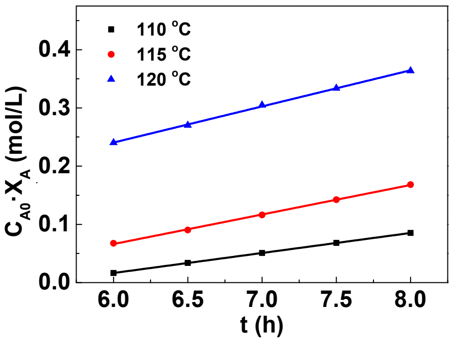

c

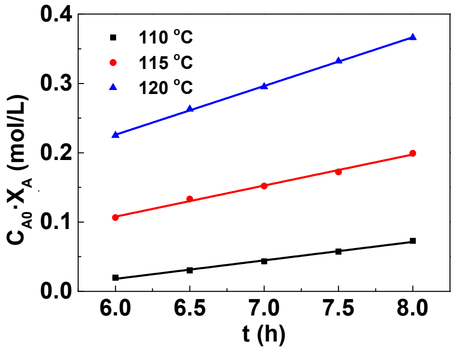

d

Figure S3. Pseudo-zero-order fits for oxidation of cyclohexane with oxygen without catalyst (a), and catalyzed by Co-TCPPNi (b), Co-TCPPFe (c) and Co-TCPPMn (d).

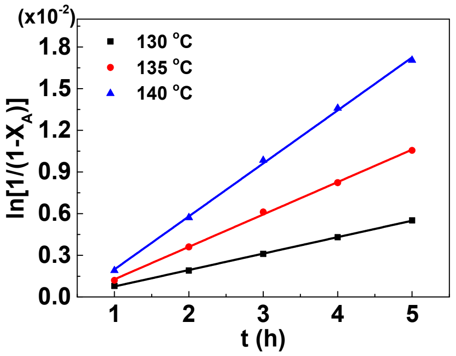

a

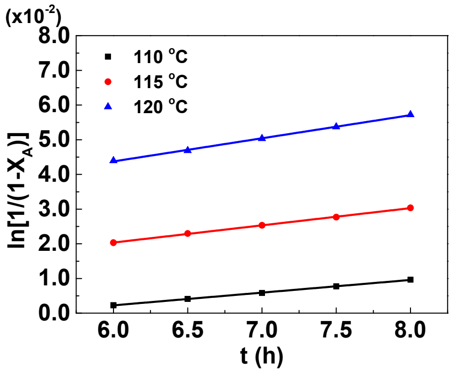

b

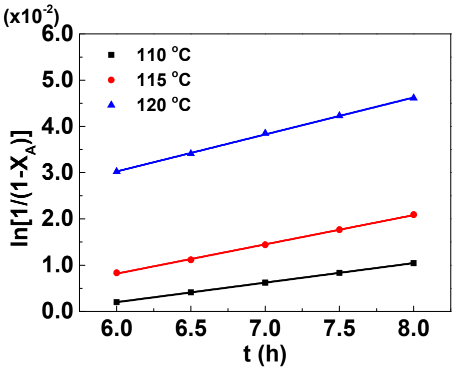

c

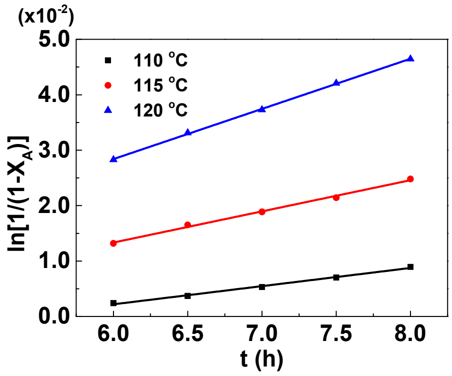

d

Figure S4. Pseudo-first-order fits for oxidation of cyclohexane with oxygen without

catalyst (a), and catalyzed by Co-TCPPNi (b), Co-TCPPFe (c) and Co-TCPPMn (d).

Table S1. The study of cycloalkanes oxygenation temperature using cyclohexane as model substrate[a].

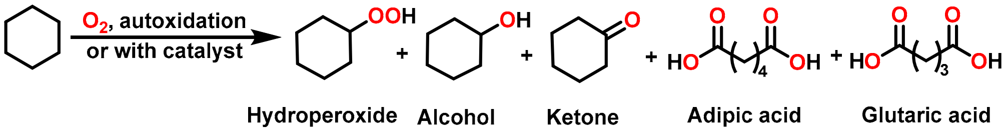

Entry	Temperature (°C)	Conversion (%)	Selectivity (%)					
			Hydroperoxide	Alcohol	Ketone	Adipic acid	Glutaric acid	Total[b]
1	100	< 0.2	/	/	/	/	/	/
2	105	< 0.2	/	/	/	/	/	/
3	110	< 0.2	/	/	/	/	/	/
4	115	< 0.2	/	/	/	/	/	/
5	120	< 0.2	/	/	/	/	/	/
6	125	0.2	85.8	/	14.2	/	/	> 99
7	130	0.4	75.7	5.5	17.8	/	/	> 99
8	135	1.0	70.0	6.7	21.6	1.7	/	98.3
9	140	6.9	17.0	33.3	30.2	18.4	1.2	80.4

[a] Autoclave reactor with a polytetrafluoroethylene-liner (100 mL), cyclohexane (200 mmol, 16.83 g), were stirred under the oxygen pressure of 1.0 MPa and the speed of 600 rpm for 8.0 h.

[b] Total selectivity to R-OOH, R-OH, and R=O as partly oxygenated products.

Table S2. Cyclohexyl hydroperoxide catalyzed oxygenation utilizing cyclooctane[a].

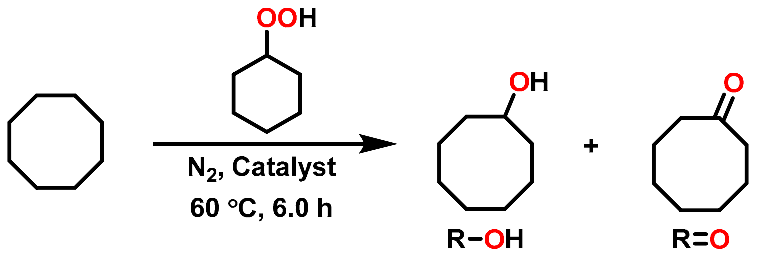

Entry	Catalysts	Conversion ($\times 10^{-2}$, %)	Selectivity (%)	
			alcohol	ketone
1	/	/	/	/
2	T(4-$COOCH_3$)PPNi(II)	1.4	7.5	92.5
3	T(4-COOH)PPNi(II)	2.2	/	> 99
4	$Co(OAc)_2$	6.3	75.2	24.8
5	T(4-$COOCH_3$)PPNi(II)&$Co(OAc)_2$	8.0	12.4	87.6
6	T(4-COOH)PPNi(II)&$Co(OAc)_2$	21.5	/	> 99

[a] Cyclohexyl hydroperoxides (1.50% in cyclohexane, mol/mol, 2.1000 g), cyclooctane (10.0 mmol, 1.1220 g) were stirred at 60 °C under the atmosphere of N_2 and the speed of 600 rpm for 6.0 h in a glass tube (25 mL). Metalloporphyrins (2.4×10^{-2}%, mol/mol), $Co(OAc)_2$ (1.0 mg/mmol) as catalysts.

Table S3. The pseudo-zero-order kinetic parameters in oxidation of cyclohexane[a].

Entry	Catalysts	Temperature (°C)	k ($L\cdot mol^{-1}\cdot h^{-1}$)	R^2
1	/	130	0.00936	0.99960

2		135	0.01825	0.99890
3		140	0.02949	0.99860
4		110	0.02986	0.99933
5	Co-TCPPNi	115	0.03936	0.99917
6		120	0.05112	0.99892
7		110	0.03440	0.99923
8	Co-TCPPFe	115	0.05074	0.99907
9		120	0.06210	0.99886
10		110	0.02668	0.99367
11	Co-TCPPMn	115	0.04488	0.99416
12		120	0.07036	0.99934

[a] Autoclave reactor with a polytetrafluoroethylene-liner (100 mL), cyclohexane (200 mmol, 16.83 g), were stirred at setting temperature with catalyst (0.08 mg/mmol, 16 mg) under the oxygen pressure of 1.0 MPa and the speed of 600 rpm for 8.0 h.

Table S4. The pseudo-first-order kinetic parameters in oxidation of cyclohexane[a].

Entry	Catalysts	Temperature (ºC)	k ($L\cdot mol^{-1}\cdot h^{-1}$)	R^2
1		130	0.00118	0.99960
2	/	135	0.00233	0.99900
3		140	0.00382	0.99880
4	Co-TCPPNi	110	0.00366	0.99930

5		115	0.00496	0.99918
6		120	0.00667	0.99878
7		110	0.00422	0.99970
8	Co-TCPPFe	115	0.00633	0.99902
9		120	0.00800	0.99898
10		110	0.00328	0.99369
11	Co-TCPPMn	115	0.00563	0.99418
12		120	0.00905	0.99938

[a] Autoclave reactor with a polytetrafluoroethylene-liner (100 mL), cyclohexane (200 mmol, 16.83 g), were stirred at setting temperature with catalyst (0.08 mg/mmol, 16 mg) under the oxygen pressure of 1.0 MPa and the speed of 600 rpm for 8.0 h.

Table S5. The pseudo-second-order kinetic parameters in oxidation of cyclohexane[a].

Entry	Catalysts	Temperature (ºC)	k (L·mol^{-1}·h^{-1})	R^2
1		130	0.00015	0.9996
2	/	135	0.00030	0.9991
3		140	0.00049	0.9989
4		110	0.00045	0.99924
5	Co-TCPPNi	115	0.00063	0.99917
6		120	0.00087	0.99864
7	Co-TCPPFe	110	0.00052	0.99990

8		115	0.00079	0.99890
9		120	0.00103	0.99906
10		110	0.00040	0.99352
11	Co-TCPPMn	115	0.00071	0.99414
12		120	0.00117	0.99941

[a] Autoclave reactor with a polytetrafluoroethylene-liner (100 mL), cyclohexane (200 mmol, 16.83 g), were stirred at setting temperature with catalyst (0.08 mg/mmol, 16 mg) under the oxygen pressure of 1.0 MPa and the speed of 600 rpm for 8.0 h.

Table S6. Quenching experiments in oxidation of cyclohexane[a].

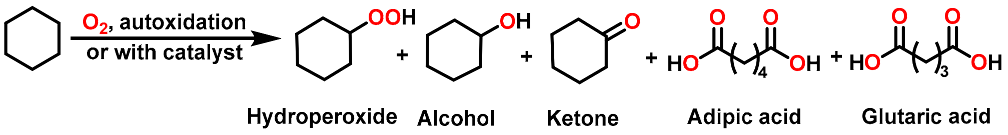

Catalyst	Temperature (°C)	Conversion (%)	Selectivity (%)					
			Hydroperoxide	Alcohol	Ketone	Adipic acid	Glutaric acid	Total[b]
-	110	0.8	23.6	25.6	50.8	/	/	> 99
	120	5.6	41.0	48.8	3.0	6.7	0.5	92.87
CBr_3Cl[c]	110	0.2	/	11.2	88.8	/	/	> 99
	120	1.0	1.1	13.9	84.8	0.17	/	99.

C_4H_9Br[d]	110	0.2	/	11.1	88.9	/	/	>99
	120	1.1	1.3	13.5	85.1	0.2	/	99.79

[a] Autoclave reactor with a polytetrafluoroethylene-liner (100 mL), cyclohexane (200 mmol, 16.83 g), were stirred at setting temperature with Co-TCPPNi (0.08 mg/mmol, 16 mg) under the oxygen pressure of 1.0 MPa and the speed of 600 rpm for 8.0 h.

[b] Total selectivity to R-OOH, R-OH, and R=O as partly oxygenated products.

[c] $CBrCl_3$ (5%, mol/mol, 5 mmol, 0.9914 g).

[d] C_4H_9Br (5%, mol/mol, 5 mmol, 0.6851 g).

Table S7. Oxygenation of cyclohexane catalyzed by double metal centers[a].

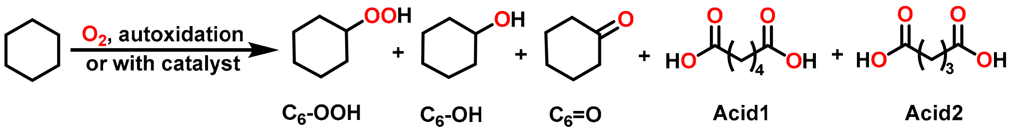

Entry	Catalysts	Conv. (%)	Selectivity (%)					
			C_6-OOH	C_6-OH	C_6=O	Acid 1	Acid 2	Total[b]
1	/	< 0.2	/	/	/	/	/	/
2	Ni-TCPPMn	0.7	59.3	19.1	21.6	/	/	>99
3	Ni-TCPPFe	0.9	61.9	/	38.1	/	/	>9

								9
4	Ni-TCPPCo	2.4	59.9	19.1	21.0	/	/	>99
5	Ni-TCPPCu	0.6	70.9	/	29.1	/	/	>99
6	Ni-TCPPZn	0.6	72.7	/	27.3	/	/	>99
7	Zn-TCPPMn	0.9	53.0	20.7	26.3	/	/	>99
8	Zn-TCPPNi	0.6	72.5	/	27.5	/	/	>99
9	Zn-TCPPFe	0.8	76.0	/	24.0	/	/	>99
10	Cu-TCPPMn	2.0	57.5	20.3	22.2	/	/	>99
11	Cu-TCPPNi	1.1	73.6	/	26.4	/	/	>99
12	Cu-TCPPFe	1.2	72.9	/	27.1	/	/	>99

[a] Catalyst (0.08 mg/mmol, 16 mg), reactor (100 mL), were stirred at 120 ^{o}C with cyclohexane (200 mmol) as substrate in the atmosphere of oxygen (1.0 MPa) for 8.0 h.

[b] Total selectivity to R-OOH, R-OH, and R=O as partly oxygenation products.

[c] T(4-COOCH$_3$)PPNi (Porp.Ni, 1.2 × 10^{-3} %, mol/mol).

[d] T(4-COOCH$_3$)PPCo&T(4-COOCH$_3$)PPNi (Porp.Co&Porp.Ni, 0.6 × 10^{-3} % + 0.6 × 10^{-3} %, mol/mol).

3. Spectra of metalloporphyrins characterization

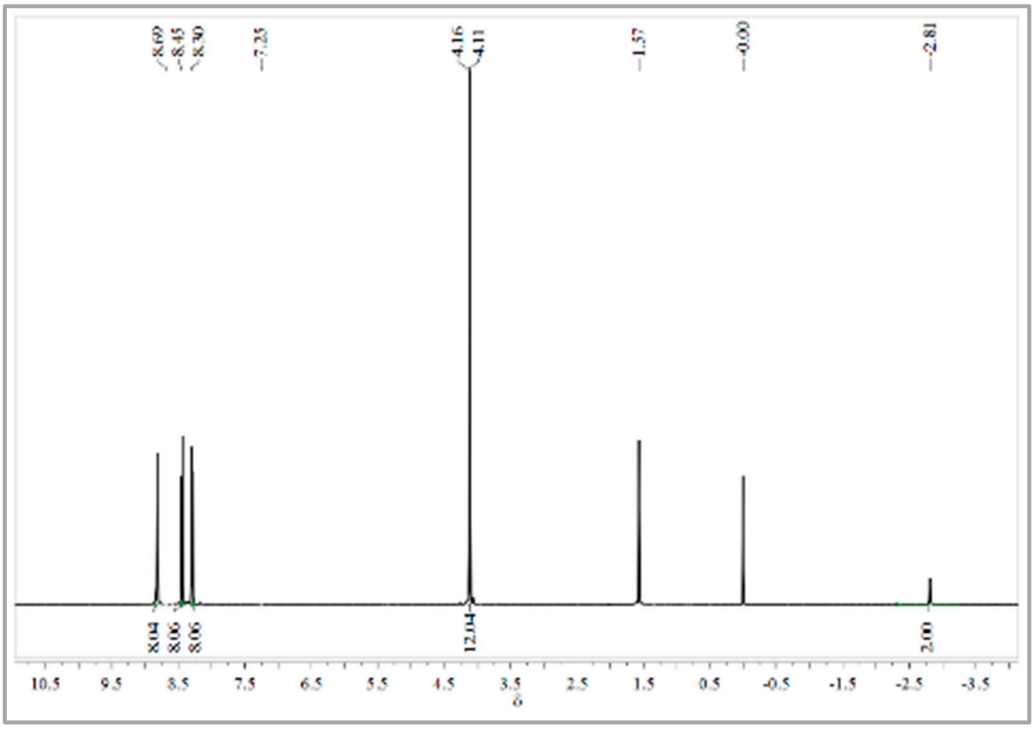

Figure S5. ^{1}H NMR spectrum of T(4-COOCH$_3$)PP

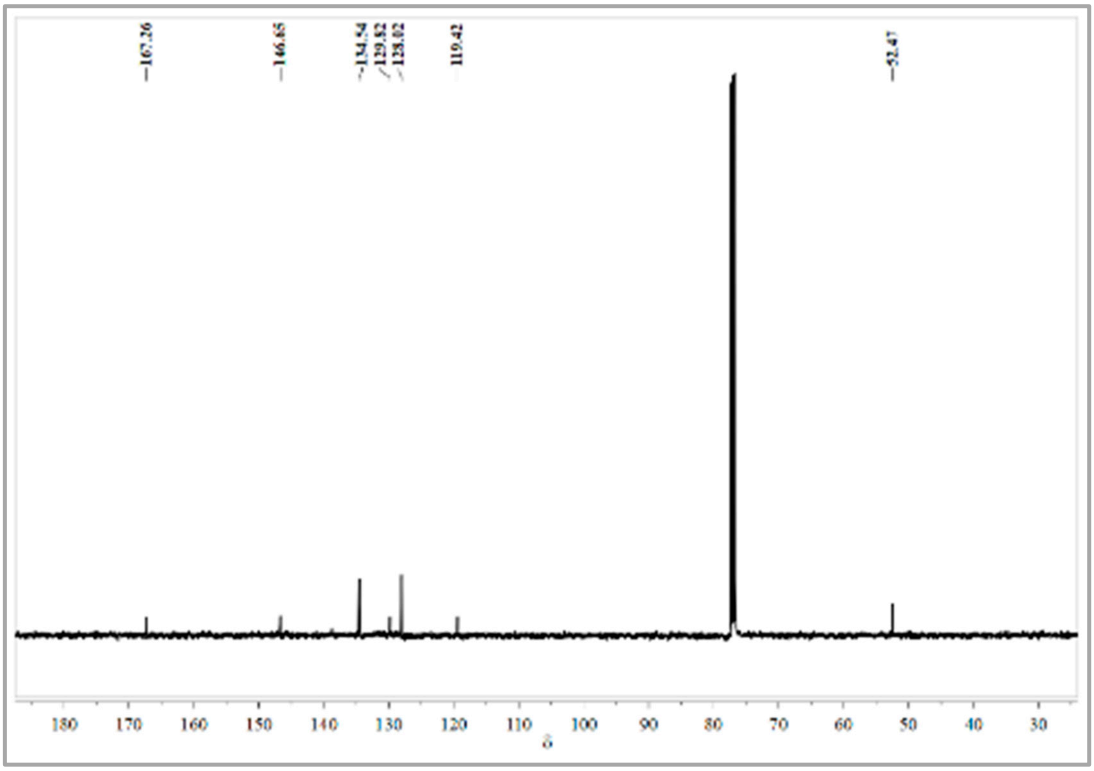

Figure S6. ^{13}C NMR spectrum of T(4-COOCH3)PP

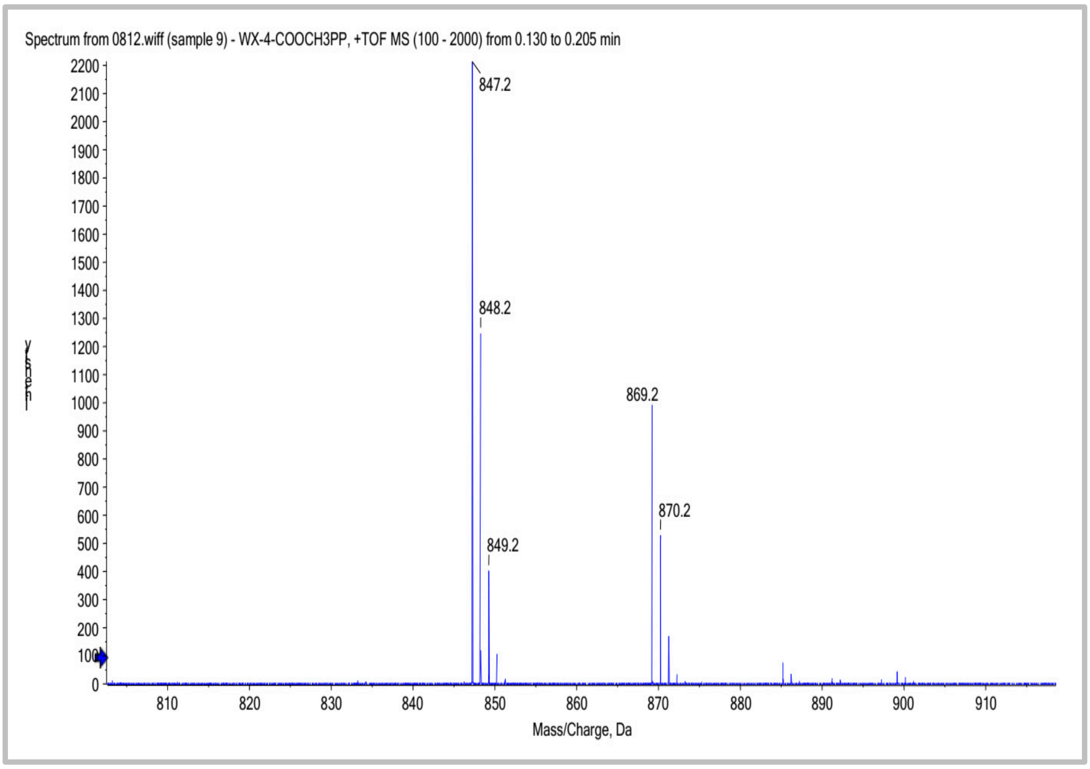

Figure S7. ESI-MS spectrum of T(4-COOCH3)PP

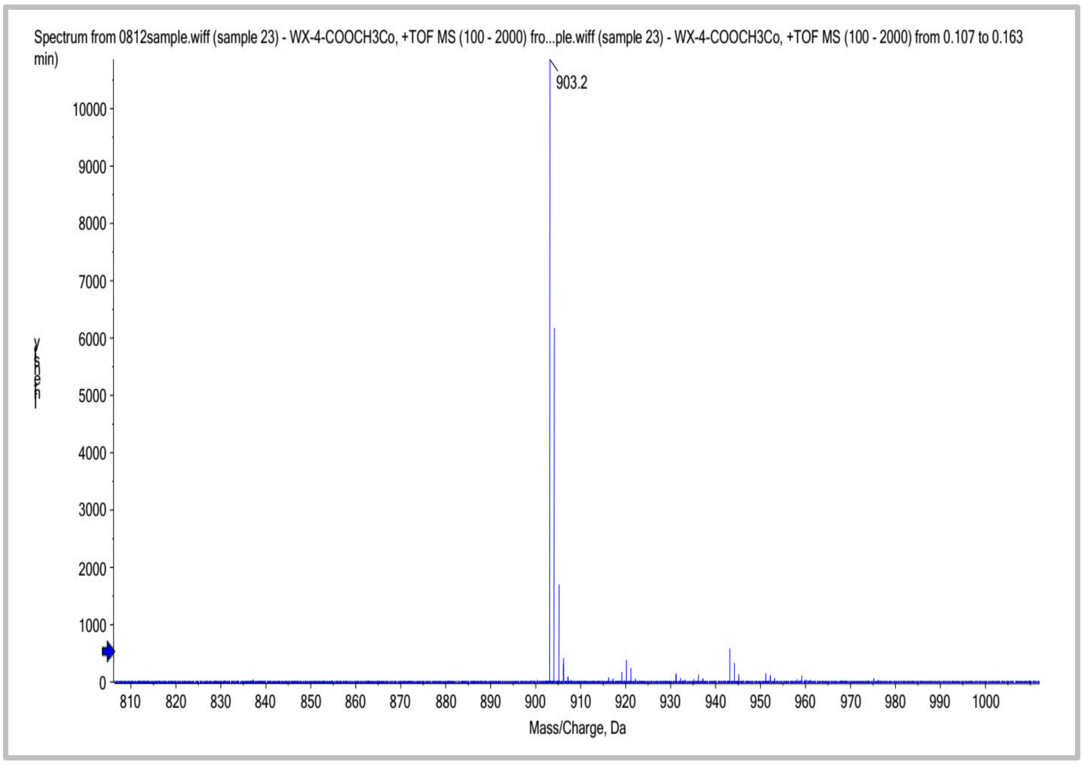

Figure S8. ESI-MS spectrum of T(4-COOCH$_3$)PPCo

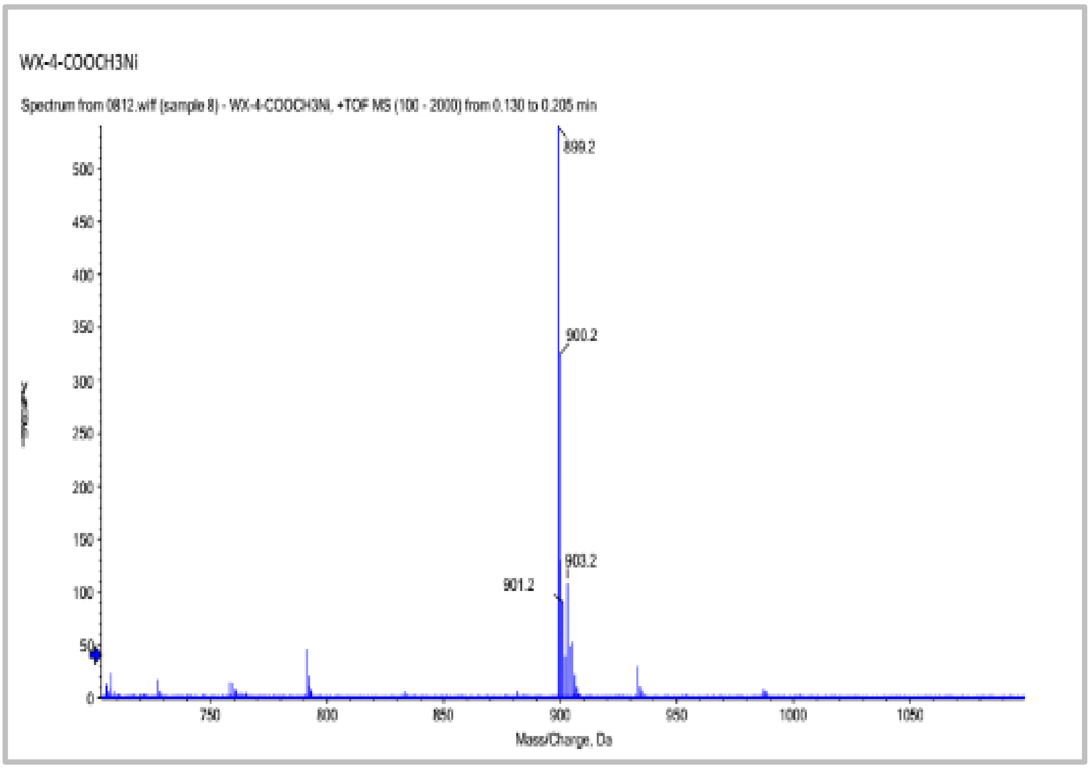

Figure S9. ESI-MS spectrum of T(4-COOCH$_3$)PPNi

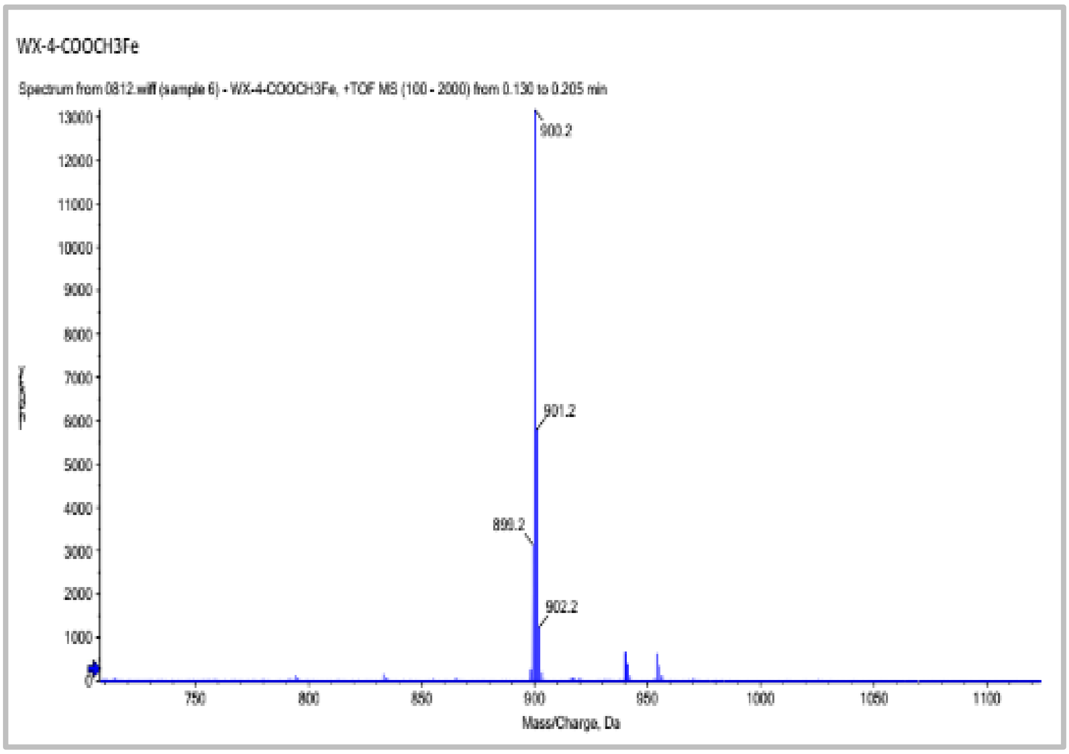

Figure S10. ESI-MS spectrum of T(4-COOCH$_3$)PPFe

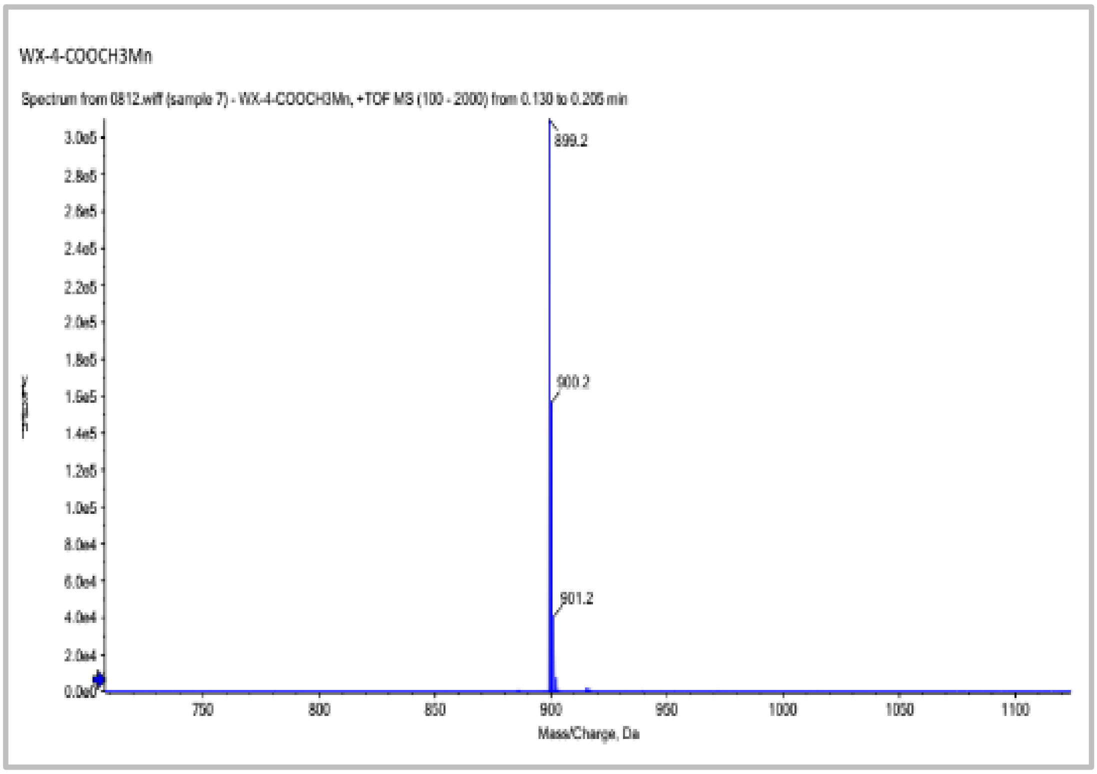

Figure S11. ESI-MS spectrum of T(4-COOCH3)PPMn

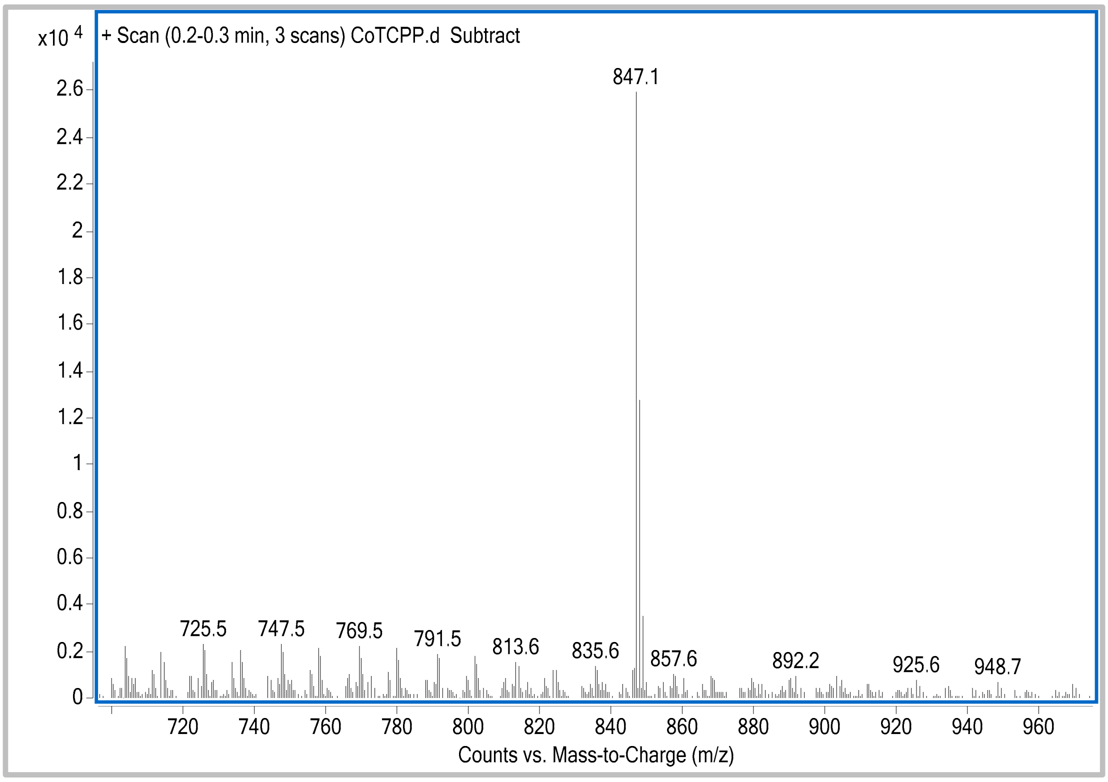

Figure S12. ESI-MS spectrum of T(4-COOH)PPCo

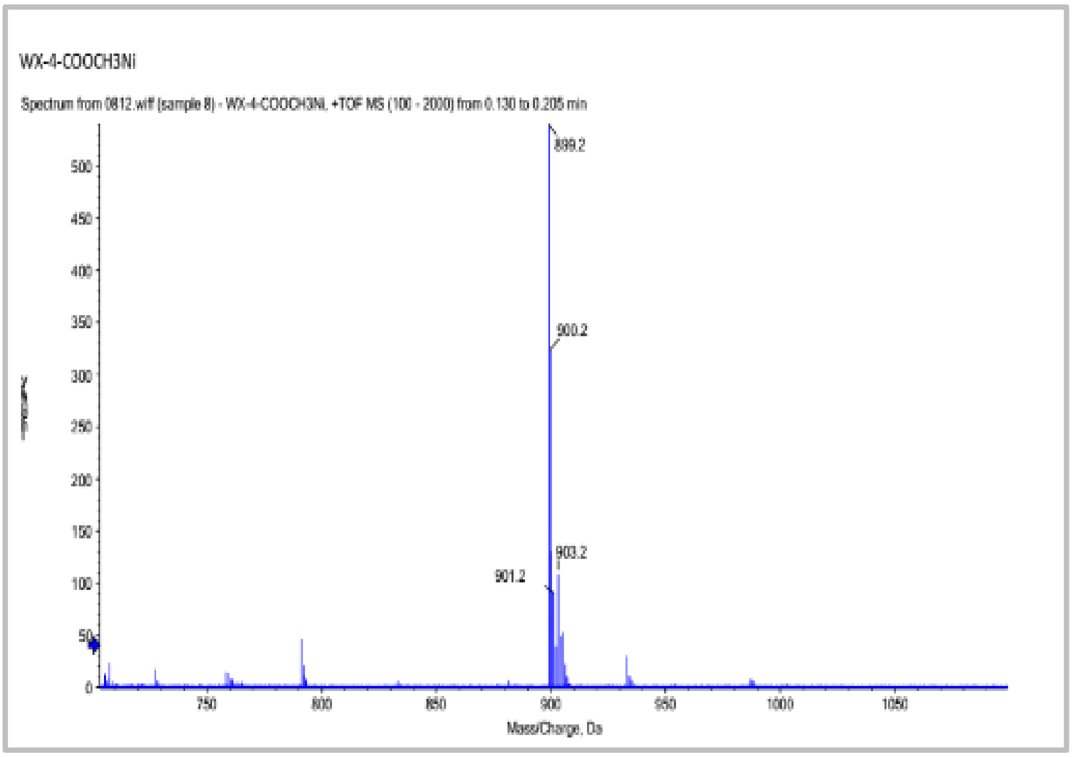

Figure S13. ESI-MS spectrum of T(4-COOH)PPNi

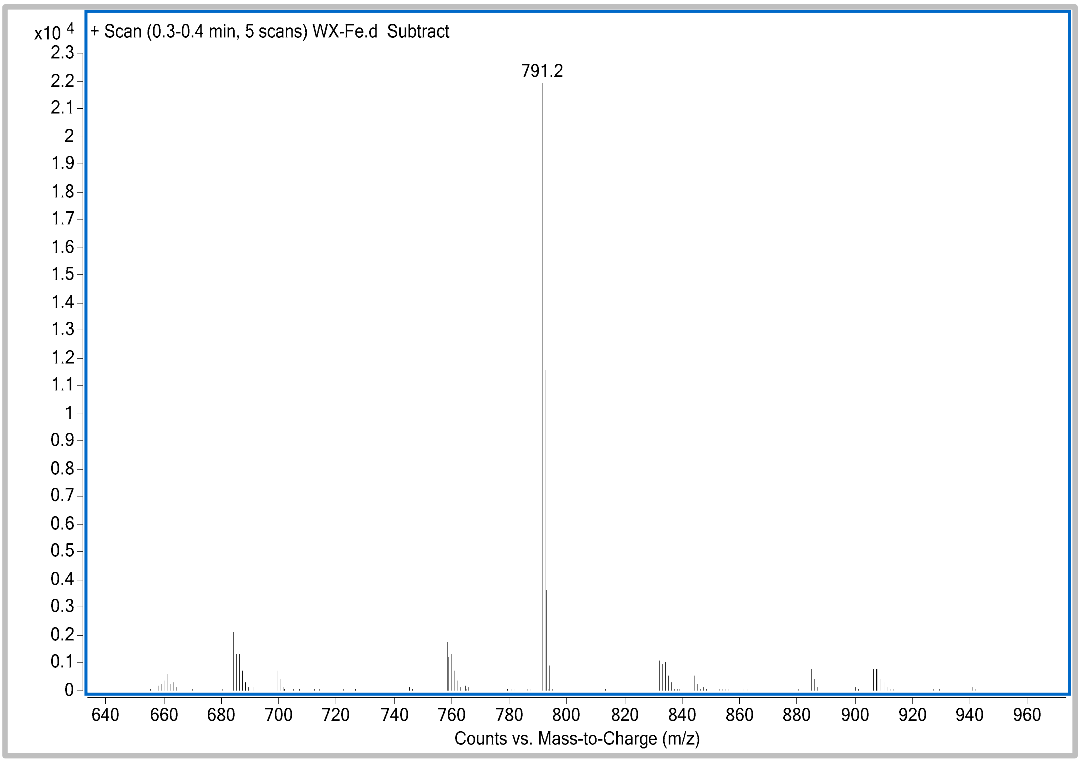

Figure S14. ESI-MS spectrum of T(4-COOH)PPFe

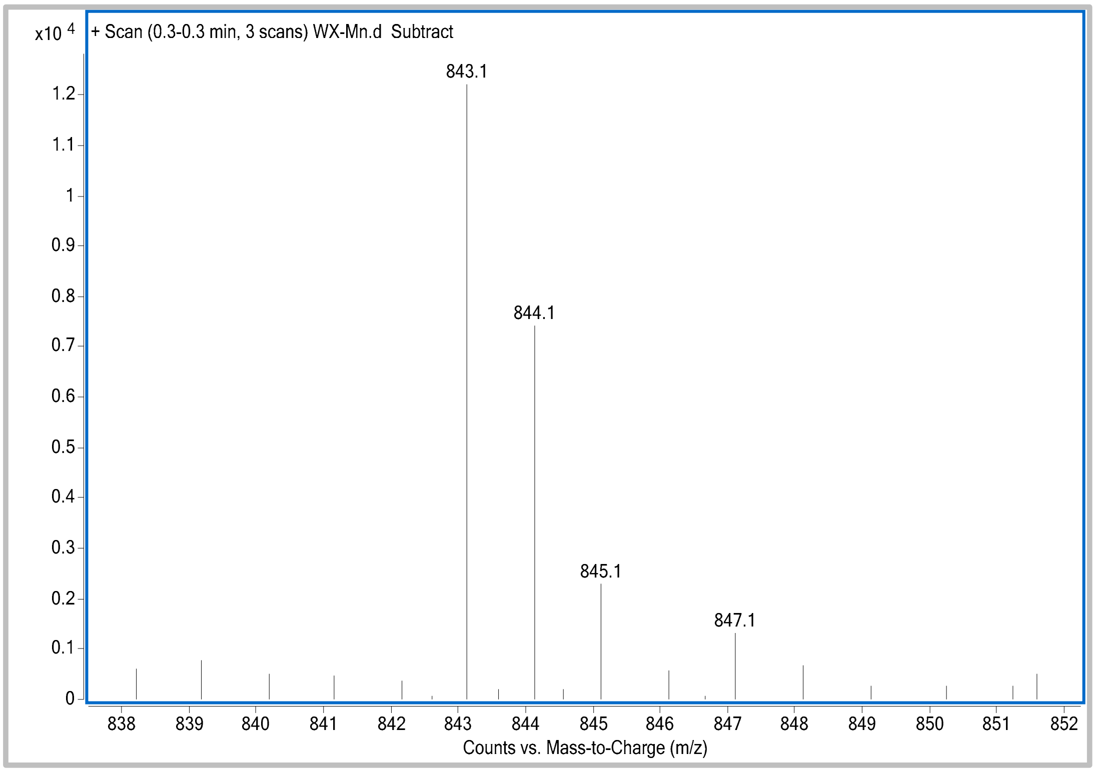

Figure S15. ESI-MS spectrum of T(4-COOH)PPMn

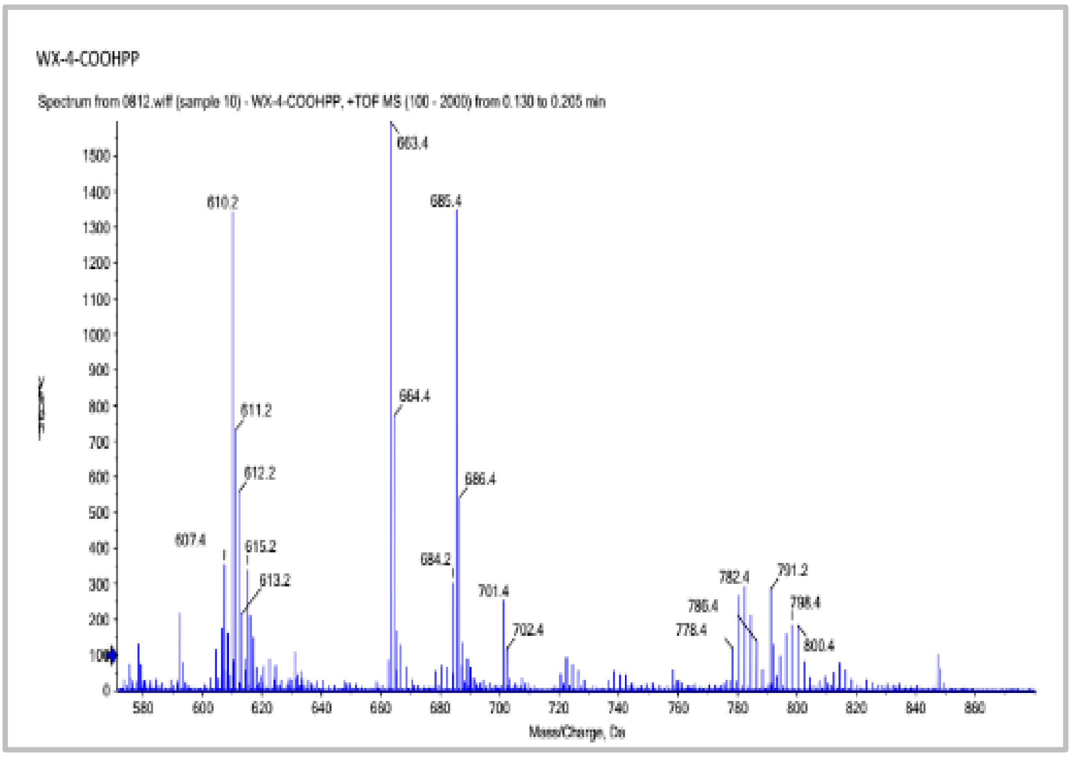

Figure S16. ESI-MS spectrum of T(4-COOH)PP

4. Spectra of radical capture

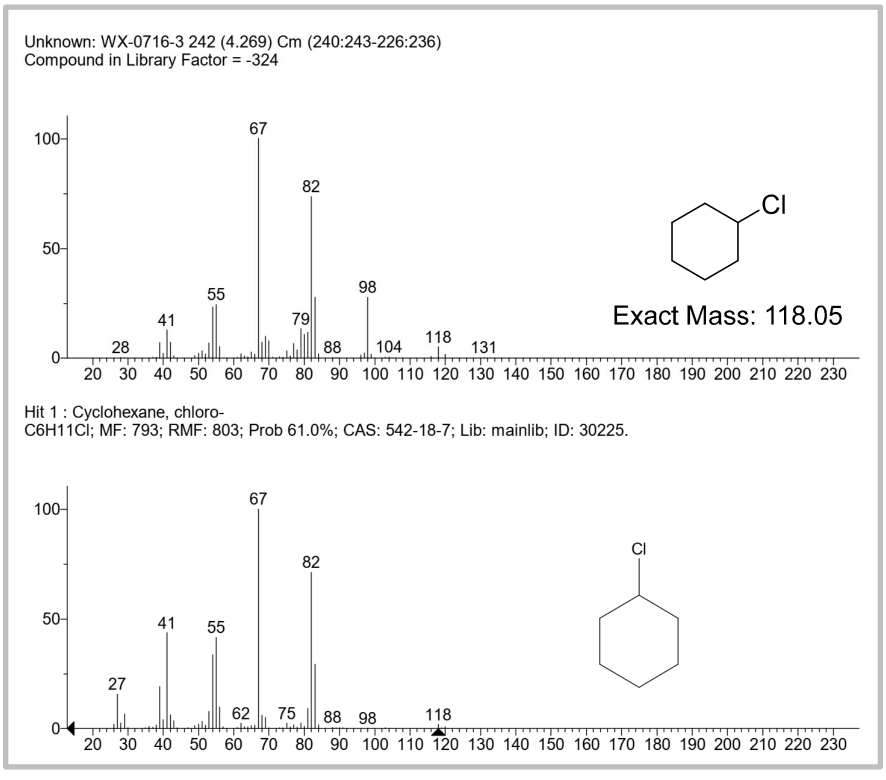

Figure S17. GC-MS spectra of quenching experiments in catalytic oxidation of cyclohexane in the presence of $CBrCl_3$-1.

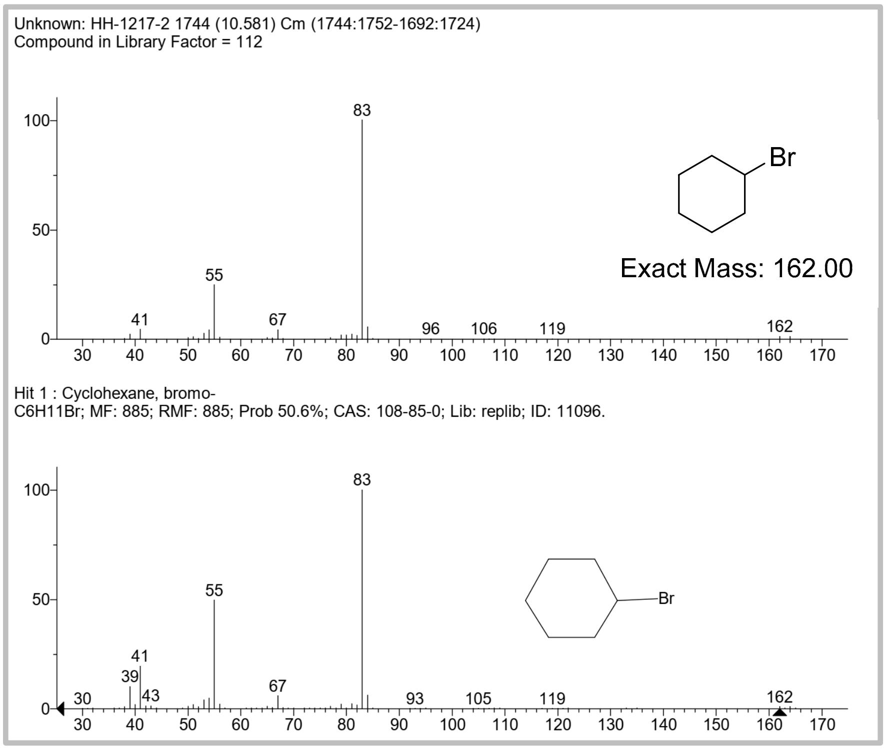

Figure S18. GC-MS spectra of quenching experiments in catalytic oxidation of cyclohexane in the presence of $(CH_3)_3CBr$-2.

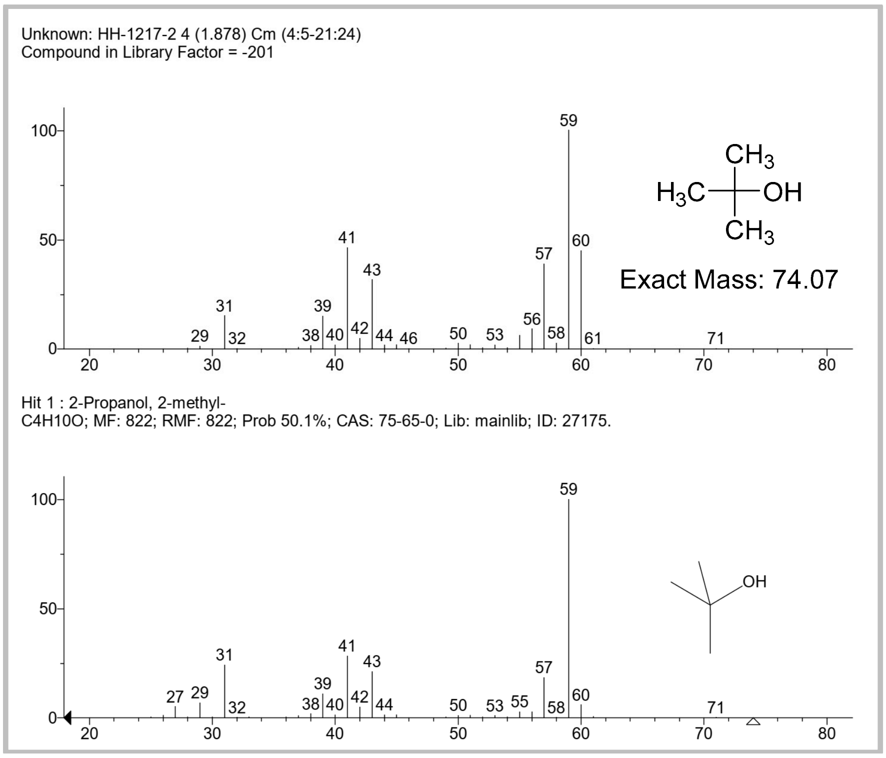

Figure S19. GC-MS spectra of quenching experiments in catalytic oxidation of cyclohexane in the presence of $(CH_3)_3CBr$-2.

5. References

[1] N. Huang, S. Yuan, H. Drake, X.Y. Yang, J.D. Pang, J.S. Qin, J.L. Li, Y.M. Zhang, Q. Wang, D.L. Jiang, H.C. Zhou, J. Am. Chem. Soc. 139 (2017) 18590-18597.
[2] D.W. Feng, W.C. Chung, Z.W. Wei, Z.Y. Gu, H.L. Jiang, Y.P. Chen, D.J. Darensbourg, H.C. Zhou, J. Am. Chem. Soc. 135 (2013) 17105-17110.
[3] D.W. Feng, Z.Y. Gu, J.R. Li, H.L. Jiang, Z.W. Wei, H.C. Zhou, Angew. Chem. Int. Ed. 51 (2012) 10307-10310.
